# Self-efficacy in relation to the use of complementary and alternative medicine, lifestyle choices and cancer aetiology

**DOI:** 10.1007/s00432-021-03857-3

**Published:** 2021-11-23

**Authors:** Lena Josfeld, Lara Krüger, Jens Büntzel, Bijan Zomorodbakhsch, Jutta Hübner

**Affiliations:** 1 Department of Hematology and Medical Oncology, University Hospital Jena, Jena, Germany; 2 Department of Otolaryngology, Südharz Klinikum Nordhausen, Nordhausen, Germany; 3 üBAG/MVZ Onkologische Kooperation Harz GbR, Goslar, Germany

**Keywords:** Oncological patients, Complementary and alternative medicine, Lay-aetiological concepts of cancer, Self-efficacy, Lifestyle choices, Patient–clinician communication

## Abstract

**Purpose:**

This survey assesses cancer patients’ etiological concepts, lifestyle choices, use of complementary and alternative medicine (CAM), and self-efficacy, as well as associations between those. It aims to find patterns which may facilitate communication and understanding between patients and physicians.

**Methods:**

353 oncological patients attending lectures on CAM answered a questionnaire. Correlations were examined and an exploratory factor analysis conducted to identify comprehensive lay-etiological concepts among a list of potential carcinogenic factors.

**Results:**

Patients considered scientifically proven agents as well as other non-carcinogenic influences to be responsible for their disease. An exploratory factor analysis yielded vague indications of possible underlying concepts but factors tend to include items that do not fit the pattern in terms of content. Higher self-efficacy correlated with healthy diet and sports, but not with use of CAM. No conclusive correlations emerged between lay-aetiological concepts and most other variables, but we found a tendency for higher self-efficacy among patients who assigned higher carcinogenic effects to tobacco and lower carcinogenic effects to fasting and physical trauma.

**Conclusion:**

Interest in CAM can arise for many reasons that are not necessarily related to self-efficacy. Lay-aetiological concepts of cancer differ significantly from scientific ones. They are complex and presumably highly individualistic. Their connection to use of CAM methods, lifestyle choices and self-efficacy should be explored in more detail. Patient information and communication with clinicians need to address cancer patients’ individual aetiological concepts to further patient’s understanding not only of their diagnosis but also of the treatment as well.

## Introduction

Complementary and alternative medicine (CAM) is often used by cancer patients (Huebner et al. 2014a; Micke et al. 2009). CAM comprises a vast group of different methods and techniques such as biological-based methods (e.g. micronutrients, other supplements, diets), holistic systems (e.g. homeopathy), body-centred techniques (e.g. massage), mind–body methods (e.g. yoga, meditation) and energy-based methods (e.g. healing touch, Reiki).

Some data exist on the aims of cancer patients while using CAM. The most often reported are to strengthen the body or the immune system, to reduce side effects of cancer treatment or to take an active part in the treatment (Huebner et al. 2014a, b). Additionally, cancer patients more or less explicitly state that they are looking for methods to combat the disease or to not miss any chance.

Patients taking an active part during treatment is one main motif for physicians and psychologists to support patients using CAM. On the other hand, risks from interactions and side effects caused mainly by biological-based CAM have to be considered (Firkins et al. 2018; Loquai et al. 2016; Zeller et al. 2013).

To improve counselling on CAM for cancer patients, it is necessary to better understand which believes and motivations drive patients to use these methods. Patients’ personal understanding of how and why they developed cancer and, consequently, how it can be cured and prevented in future, may play an important role in the decision if and which CAM methods are being used. To investigate this further, we formulated the following hypotheses to examine.

1. There is an association between lay-aetiological concepts of cancer and the type of CAM used.
  Lay-aetiological concepts on the development of cancer are quite different from modern molecular understanding. In fact, stress and mental traumata are most often cited by patients as reasons for cancer, while unhealthy lifestyle, smoking and alcohol are only named by a small minority (Huebner et al. 2014a, b; Paul et al. 2013). A variety of CAM methods may be employed to address issues which patients believe to be responsible for the development and persistence of cancer.
2. Patients with higher self-efficacy are more likely to use CAM.
  Self-efficacy is a concept that is related to an engagement in a healthy lifestyle (Ram and Laxmi 2017). Self-efficacy has been defined as a person’s assessment of their ability to cope with a (difficult) situation, based on their own skills and considering current circumstances (Bandura 1994). The perception of one’s own ability to take an active influence in one’s own life is likely to encourage people to adapt a healthy lifestyle if they wish for their health to improve. Using CAM methods can be viewed as another possibility for patients to actively take part in improving their health if they do not wish to leave everything in the hands of their treating physician(s).

Accordingly, the hypothesis that higher self-efficacy is associated with a higher interest of cancer patients in CAM is widespread. In contrast, preliminary data from our own surveys have not found such an association (Ebel et al. 2015).

## Methods

### Participants

We recruited oncological patients from a series of lectures on complementary and alternative medicine. These were held in 20 different German cities, running from May through October 2019. They were gratuitous and addressed cancer patients and their caregivers. Lectures were presented by the working group Prevention and Integrative Oncology of the German Cancer Society and held by a specially trained oncologist in non-expert language, providing evidence-based information. Attendees received a questionnaire at the end; participation was voluntary and anonymous. Only data from current or previous oncological patients was included in the analyses.

### Questionnaire

The questionnaire assembled for this study contained five main sections.

1. Demographic data: Age, gender, education level, type of cancer, and year of first cancer diagnosis.
2. Lifestyle: We first asked for participants’ subjective assessment of their dietary habits (healthy, unhealthy, normal, vegetarian, vegan etc.) to later correlate it with their subjective assessment of nutritional factors’ influence on cancer. Multiple answers were possible. Physical activity was assessed before and after the diagnosis, and differentiated between everyday activity (e.g. going for a walk, walking to work, to the shop etc.) and weekly exercising/sports. In accordance with current recommendations for physical activity and exercise (World Cancer Research Fund 2019), answers were grouped into 0–30 min vs. over 30 min of routine activity per day, and 0–2 h vs. over 2 h of sports per week.
3. CAM: In addition to general interest in and use of CAM, participants were given a list of 21 methods to indicate which ones they have used since their first cancer diagnosis. The list was compiled of methods which had previously been found to be the most frequently used in Germany (Huebner et al. 2014a, b; Paul et al. 2013).
4. Self-efficacy: Assessed via the German Self-Efficacy Scale Short Form (ASKU) which consists of three statements on the perceived ability of dealing with difficult situation (Beierlein et al. 2012). Statements were rated on a 5-point Likert scale ranging from “not at all true” to “exactly true”. Mean values were used for analysis.
5. Lay-aetiology: Participants were asked to rate their perceived/assumed influence of a list of potential carcinogenic agents on a 5-point Likert scale ranging from “not at all” to “very high”, with an additional category “I don’t know”. The listed agents were a mixture of scientifically proven carcinogens from the list of the International Agency for Research on Cancer (IARC) (International Agency for Research on Cancer 2019), agents prominently discussed in lay media, and treatment used in standard cancer therapy or complementary and alternative medicine (for individual items see Table 2).

Several patients tested the questionnaire for feasibility and comprehensibility prior to the survey. The survey was approved by the ethics committee at the University Hospital of the Friedrich Schiller University at Jena (ethics no. 2019-1394). The procedures used in this study adhere to the tenets of the Declaration of Helsinki.

### Statistics

IBM SPSS Statistics Version 26 was used for statistical analyses. Correlations were explored via Spearman correlation, cross tables and chi-square tests, depending on the scale of measurement; Fisher’s exact test was used were data did not meet the requirements for a chi-square test. A *p* value below 0.05 was considered significant.

An exploratory factor analysis with maximum likelihood estimation and varimax rotation was conducted over part five of the questionnaire, to extract groups of influencing factors which patients may view similarly or as belonging together.

## Results

### Demographic data

The questionnaire was returned by 353 oncological patients. The demographic data of participants in the survey are summarized in Table 1. Age was expectedly high with a mean between 60 and 69, and only 12.7% were younger than 50. The large majority of participants were female, the most common type of cancer was breast cancer. Most patients had been diagnosed between one and nine years previous.

**Table 1:** Demographic data of participants (*N* = 353)

	*n* (% of valid answers)
Age	*N* = 353
< 50	45 (12.7)
50–59	90 (25.5)
60–69	128 (36.3)
70–79	77 (21.8)
80+	13 (3.7)
Gender	*N* = 353
Female	248 (70.3)
Male	105 (29.7)
Education level	*N* = 350
No qualification	5 (1.4)
Secondary school qualification (9 or 10 years)	93 (26.5)
University entrance diploma	11 (3.1)
Vocational training	126 (36.0)
University/polytechnical degree	115 (32.9)
Type of cancer	*N* = 380 (multiple answers)
Breast cancer	161 (42.37)
Gastrointestinal cancer	64 (16.84)
Prostrate cancer	31 (8.16)
Lung cancer	15 (3.95)
Leukemia/Lymphoma	27 (7.11)
Gynaecological cancer	20 (5.26)
Malignant melanoma	22 (5.79)
Other	40 (10.53)
Year of first diagnosis	*N* = 353
Before 2000	36 (10.2)
2000–2009	45 (12.7)
2010–2018	186 (52.7)
2019	86 (24.4)

### Lifestyle

#### Dietary habits

**Fig. 1:**
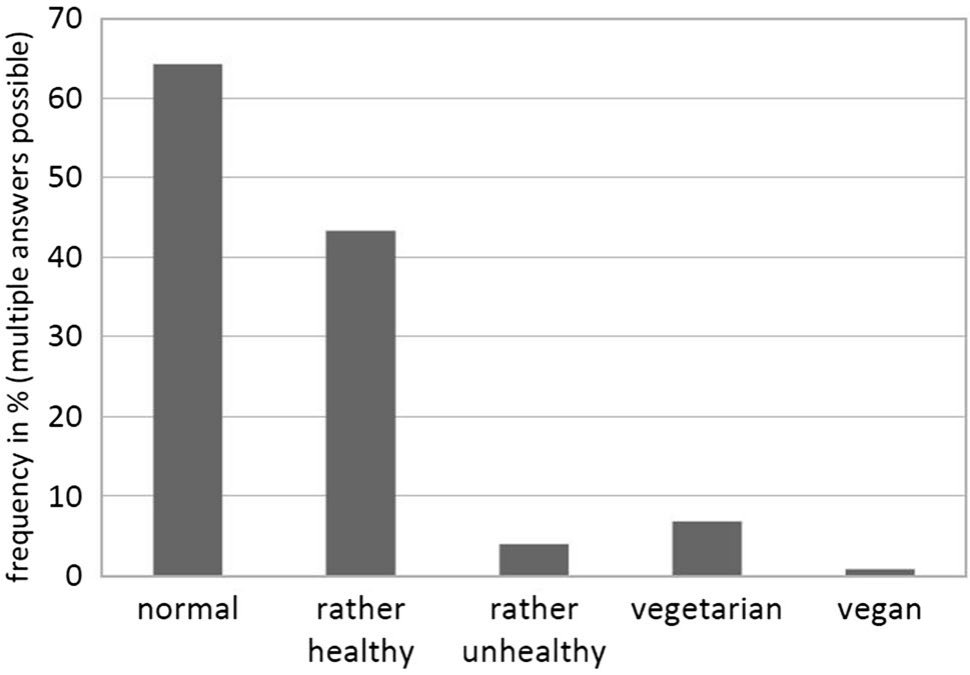
Dietary habits as described by participants (*N* = 353)

The majority of participants described their dietary habits as “normal” (64.3%), 43.3% described them as “rather healthy”, very few as “rather unhealthy” (4%), “vegetarian” (6.8%), or “vegan” (0.8%; see Fig. 1).

#### Physical activity

Prior to their diagnosis, the majority of participants had more than 30 min of daily routine activity like going for a walk or walking to work etc. (85.7%, *N* = 335) and did sports for over 2 h per week (57.1%, *N* = 315).

After being diagnosed with cancer, the number of patients with more than 30 min of daily activity decreased but remained the majority (70.9%, *N* = 330). Sports decreased as well so that only 47.1% (*N* = 312) now did more than 2 h per week.

Daily routine activity correlated positively with doing sports both before the first cancer diagnosis (*χ*^2^(1) = 10.559, *p* = 0.001) and at the time of the survey (*χ*^2^(1) = 43,462, *p* < 0.001). Physical activity prior to diagnosis correlated positively with physical activity after diagnosis (daily routine activity: *χ*^2^(1) = 26.669, *p* < 0.001); sports: *χ*^2^(1) = 43.798, *p* < 0.001).

### Use of complementary and alternative medicine

**Fig. 2:**
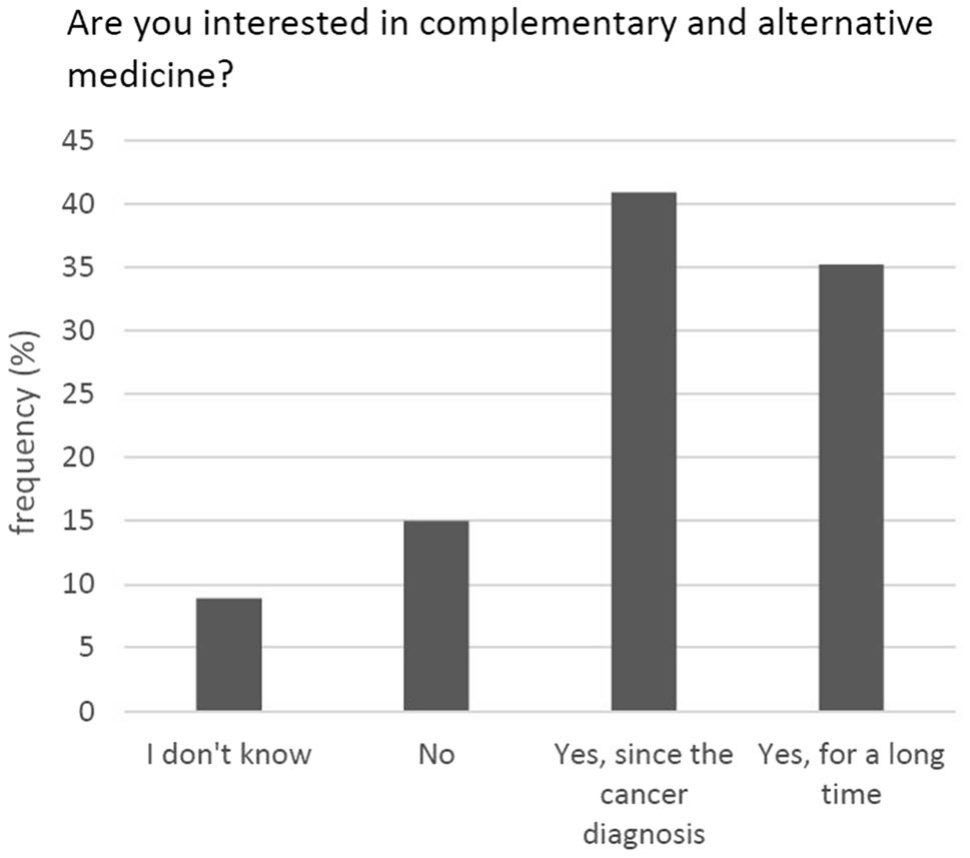
Interest in CAM (*N* = 347)

**Fig. 3:**
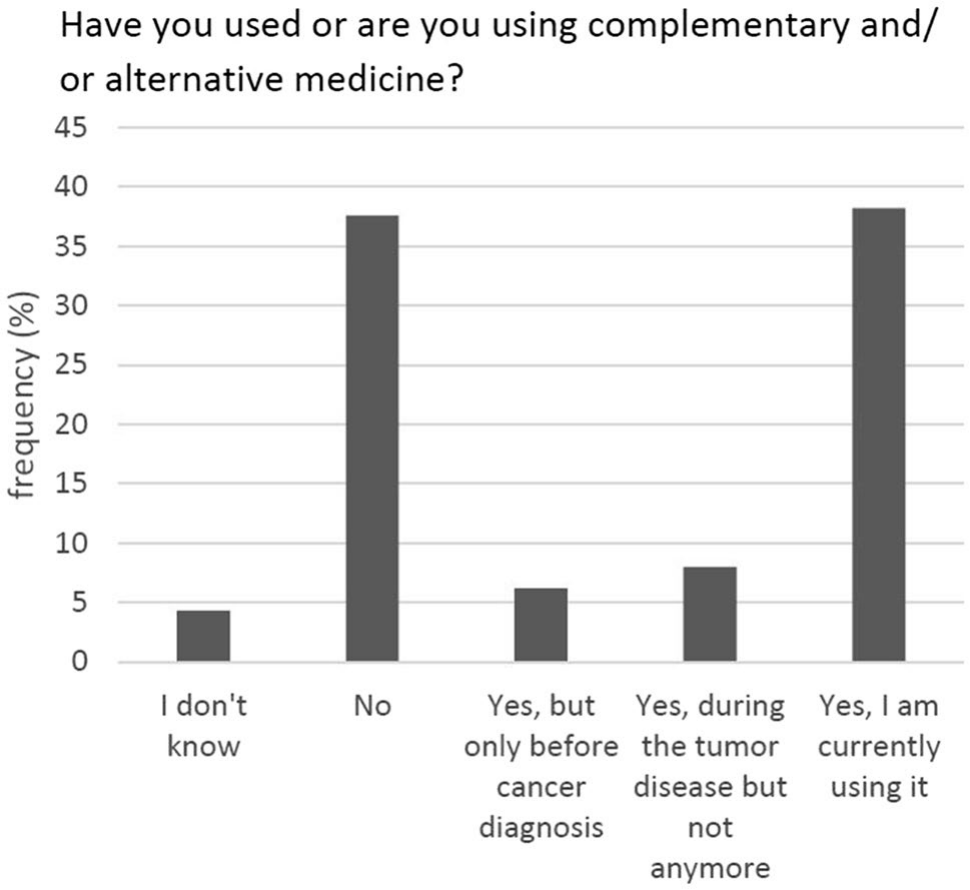
Use of CAM (*N* = 338)

A total of 74.8% of the participants reported an interest in CAM—either since their tumour diagnosis or before (Fig. 2). Fifty percent had used or were currently using such methods (Fig. 3).

**Fig. 4:**
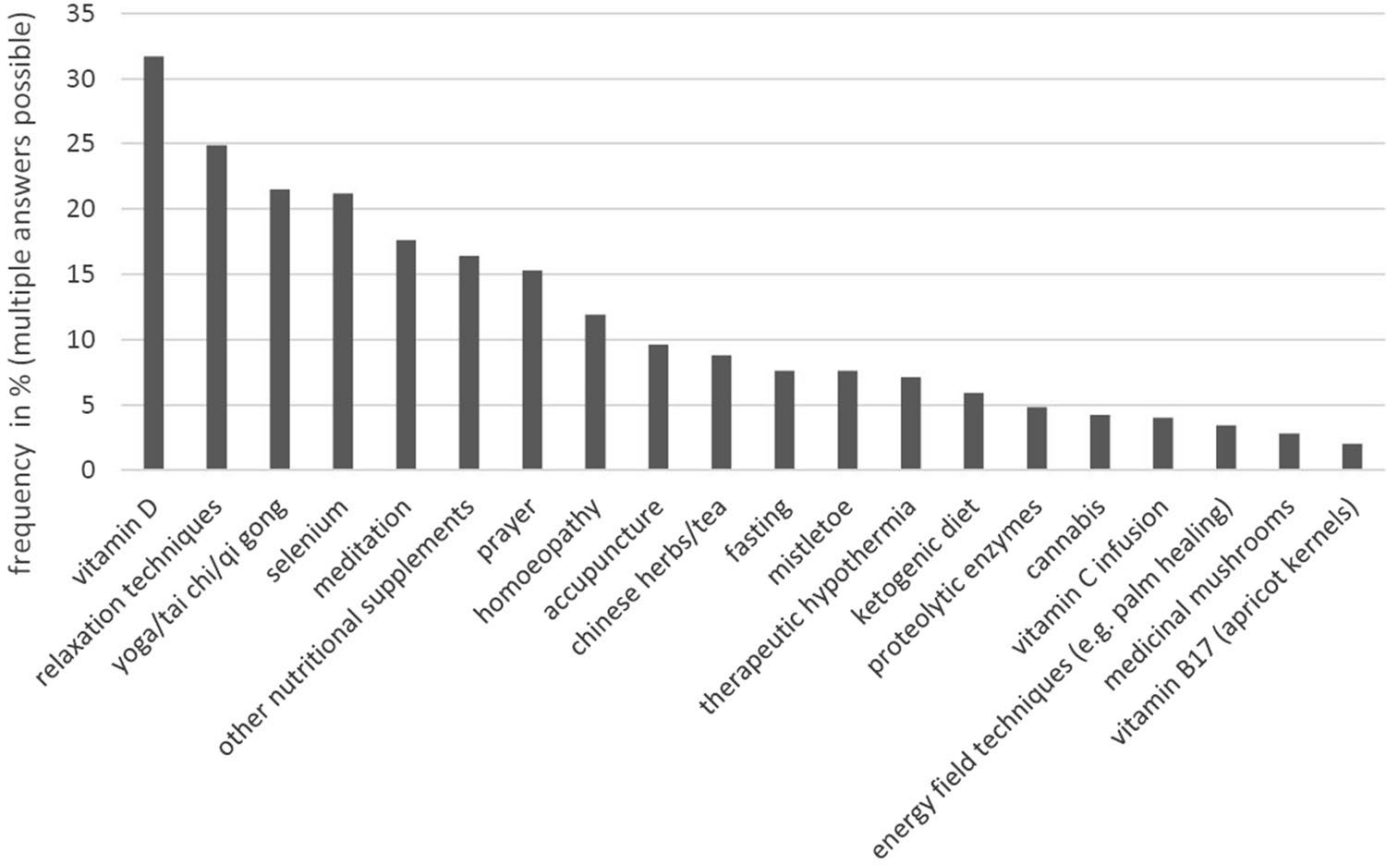
Use of individual CAM methods (*N* = 353)

Figure 4 shows the use of different CAM methods in this study. Of the most frequently used, by over 20% of participants, two were nutrition supplements (vitamin D 31.7%; selenium 21.2%) and two strategies for physical balance (relaxation techniques 24.9%; yoga/tai chi/qi gong 21.5%).

### Self-efficacy

Overall self-efficacy of the study’s sample ranged between 1 and 5 with a mean value of 3.85 and a standard deviation of 0.72. This result lies only slightly below the mean of 4.0 (SD 0.74) of the norm sample over all age groups (Beierlein et al. 2012). This corresponds with previous findings that self-efficacy in cancer patients in general does not deviate significantly from the norm (Hinz et al. 2019; Melchior et al. 2013; Thieme et al. 2017; Thieser et al. 2021).

### Lay-aetiologic concepts

**Fig. 5:**
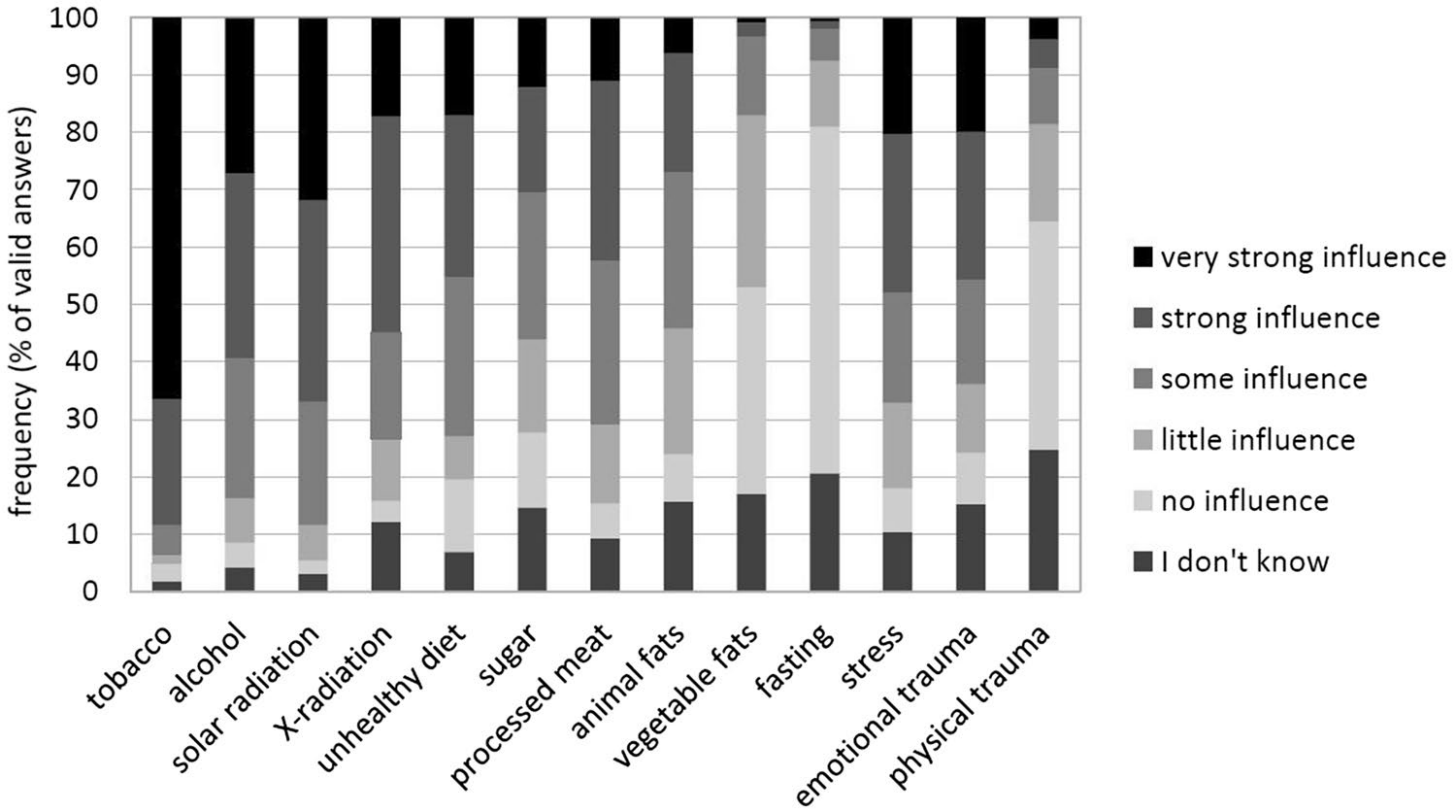
Lay-aetiological concepts (*N* = 353)

The highest-scoring factors regarding their perceived carcinogenic influence were tobacco (88.5% assumed it to be “highly” or “very highly carcinogenic”), solar radiation (66.8%), alcohol (59.4%), and X-radiation (54.8%). Factors with over 40% of answers describing them as “highly” or “very highly carcinogenic” were stress (48.1%), emotional trauma (45.8%), unhealthy diet (45.3%), and processed meat (42.3%). Figure 5 displays the distribution of answers for a variety of assessed factors (Hinz et al. 2019; Melchior et al. 2013; Thieme et al. 2017; Thieser et al. 2021).

There was considerable dropout towards the end of the questionnaire resulting in few complete answers on carcinogenic effects. A listwise deletion of missing data during the exploratory factor analysis for carcinogenic influences reduced the sample size to *N* = 51.

The exploratory factor analysis yielded relatively few applicable results. A cut-off for the measure of sampling adequacy (MSA) at 0.5 as literature suggests (Ludwig-Mayerhofer 2004) excluded such important items as tobacco, alcohol, processed meat, solar radiation and various others. Setting the cut-off to 0.4 for exploratory purposes yielded the resulting six to seven factors for each category presented in Table 2. In most cases, the results were not distinct, as many items loaded onto several factors to a similar extend.

**Table 2:** Resulting factors for patients’ lay-aetiological concepts (*N* = 51)

Factor	Carcinogenic agents loading on factor
1	Cosmic forces, another person’s wish, surgery, energy fields^2^
2	Mistletoe therapy^2^, vitamin B17^2^, homeopathy, vitamins, vegetable fats,
3	Emotional trauma, stress, chemotherapy^2^
4	Animal fats, sugar, unhealthy diet^2^, plant extracts, fasting
5	Viruses^2^, X-radiation^2^, hormonal contraception
6	Alcohol^2^, tobacco^2^, physical trauma^2,3^
	Processed meat^1^, solar radiation^1^, physical activity^1^

^1^Items excluded due to measure of sampling adequacy < 0.4

^2^Loading on factor < |0.6|

^3^Negative loading on factor

### Correlations

#### Self-efficacy and lifestyle

“Eating healthy” was associated with higher self-efficacy, though the eta coefficient was low (0.225). Similar associations were found between higher self-efficacy and sports both prior to tumour diagnosis (eta = 0.205) and currently (eta = 0.238).

#### Self-efficacy and CAM usage

No correlations could be found between self-efficacy and interest in CAM or self-efficacy and general use of CAM. Among the 21 different CAM methods, only therapeutic hyperthermia yielded a significant result (Fisher’s exact test: *p* = 0.015): participants who had used hyperthermia tended towards a higher self-efficacy than those who had not. Due to the large number of tests that had been run increasing the risk of an alpha error, this result has to be considered as a possible statistical artefact.

#### Self-efficacy and lay-aetiology

Of all 346 patients, those with higher self-efficacy were more likely to assume higher carcinogenic influence of tobacco (*r*_s_ = 0.117, *p* = 0.034). They were less likely to assign carcinogenic influence to fasting (*r*_s_ = − 0.227, *p* < 0.001) or physical trauma (*r*_s_ = − 0.179, *p* = 0.005). No correlations were found with any of the other items.

#### Lifestyle and lay-aetiology

Patients who described themselves as eating “rather healthy” were more likely to assume a carcinogenic influence of processed meat (*χ*^2^(5) = 20.289, *p* = 0.001), unhealthy diet (*χ*^2^(5) = 19.804, *p* = 0.001) and sugar (*χ*^2^(5) = 13.749, *p* = 0.017). Patients not describing their diet as “rather healthy” were more likely to be unsure about the influence of animal fats (*χ*^2^(5) = 20,527, *p* = 0.001). There were no significant correlations between eating healthy and the assumed influence of fasting or vegetable fats. Vegetarians were more likely to assume a higher carcinogenic influence of processed meat (*χ*^2^(5) = 15,466, *p* = 0.009).

## Discussion and conclusion

### Discussion

Due to the selective group of participants, there is an expectedly high number of patients with personal interested in CAM (nearly 80%), half of them having used or currently using CAM. Still, both numbers are in line with national and international publications (Bauer et al. 2018; Horneber et al. 2012; Huebner et al. 2014a; Molassiotis et al. 2005) and underline the growing importance of the topic among oncological patients.

Very different CAM methods are used, most frequently so-called biological-based methods as micronutrients but also herbs which may be in some cases beneficial but also put users at risk of genuine side effects and interactions (Firkins et al. 2018; Zeller et al. 2013). Mind–body techniques as yoga, tai chi or qi gong are taught in quite different styles with more or less meditative and physically active components.

Previous studies have shown that patients pursue different goals when using CAM: strengthening one’s own immune system and/or general energy levels, alleviating side effects, combatting the cancer or taking an active part in the treatment (Huebner et al. 2014b). While for most of the patients’ goals, there is little evidence of CAM being effective in those regards, the evidence for physical activity on side effects of cancer treatments, quality of life and survival is overwhelming (Friedenreich et al. 2019). For nutrition as the second lifestyle factor, data are growing which highlight the importance of a healthy nutrition and the avoidance of loss of weight and sarcopenia (Arends et al. 2017).

In contrast, our data show that not even half of the patients comply with the recommendations of the national cancer guideline program (Leitlinienprogramm Onkologie 2021) and even reduce physical activity after their diagnosis. This, in part, may be due to a lack of information during the treatment (Höh et al. 2018). Yet, most patients are interested in doing sports (Roth et al. 2020), and users of CAM are more physically active than non-users (Loquai et al. 2017). Moreover, most patients rate their diet as “normal” which most probably means western style and not healthy. One interpretation would be that they are indeed not eating as healthy as recommended. Another interpretation would be that some patients—perhaps especially those of higher health consciousness—are eating healthy enough but are aware that they could still do better and thus do not consider their diet as particularly healthy. Using a more objective measure than a self-reported assessment of “healthy”, “unhealthy” or “normal” diet may shed more light on this phenomenon in future studies.

Accordingly, for physicians counselling cancer patients who are looking for ways to participate in the treatment process, besides an evidence-based counselling on CAM, counselling on nutrition and physical activity reconciles patients’ aims and interests and evidence. It may also be well worth for physicians to enquire what may keep patients from adapting a healthy lifestyle beyond a lack of information (e.g. the family’s lifestyle, unmentioned symptoms etc.).

Self-efficacy in our collective is quite similar to other cancer patients and the general population. No correlations could be found between self-efficacy and interest in CAM or use of CAM. This is in contrast to a widespread belief that CAM usage is a result of high self-efficacy. In fact, in a former survey, we found a similar result and even an association of CAM usage with a high external locus of control (Ebel et al. 2015). In contrast, patients with a higher self-efficacy less often suppose unhealthy nutrition being the cause of their cancer (Welter et al. 2021), and in this survey, we found an association of “eating healthy” and sports to higher self-efficacy. This may offer physicians a strategy in counselling patients who want to become active first of all on a healthy lifestyle.

In accordance with the present findings, several of our previous surveys have shown that for different patient groups, causes of cancer are different from the scientific concepts. Stress and mental trauma are among the most frequently named causes (Huebner et al. 2014b; Welter et al. 2021). In recent times, toxins—especially environmental toxins—and genes are also named (Huebner et al. 2014b). In contrast, unhealthy lifestyle is only named by a minority, even in a collective of patients with strong smoking and drinking habits (Paul et al. 2013).

In one of our former surveys, we ran a preliminary analysis on the association of lay-aetiological concepts and the type of CAM used without getting a significant or meaningful result (Ebel et al. 2015). To better understand these concepts, we decided to run a factor analysis with our new data. Unfortunately, only a small part of the patients responded to all necessary items of this part of the questionnaire. Accordingly, the interpretation is difficult and only exploratory. Factor 1 (Table 2) includes cosmic forces, another person’s wish, and energy fields and might combine unknown power. Factor 2 combines established methods of CAM in Germany as mistletoe, vitamin B17 (amygdalin, a cyanogen substance), homeopathy and vitamins. Factor 3 consists of the above-mentioned items emotional trauma, stress, while factor 4 focuses on nutrition (animal fats, sugar, unhealthy diet, plant extracts, fasting). While factor 5 includes external agents, the patient may not control (viruses, X-radiation), factor 6 lists external agents the patient may control (alcohol, tobacco). Yet, in all factors, there are agents that from a scientific point of view do not fit the underlying concept of the factor as for example surgery in factor 1, hormonal contraception in factor 5 and physical trauma in factor 6.

This is likely due to the low number of participants in this section of the questionnaire. Yet, it might also point to lay-people having concepts of diseases that are quite different from our scientific concepts. We have shown a similar scenario in a survey on non-medical practitioners, which found no scientifically based association between the diagnostic and therapeutic strategies these non-medical practitioners used (Koehl et al. 2014).

The decreasing answers to the final tables of the questionnaire (regarding carcinogenic, therapeutic and alleviating influence of various factors) may indicate patients’ difficulties with the posed questions and point to a much larger number of patients lost in understanding why they were hit by cancer and how they are supposed to cope with it now.

### Limitations

There are several limitations to our work. First of all, the setting of the survey does not allow to calculate the response rate as we did not asses the total number of lecture attendees. Moreover, we addressed a selective group with a high interest in CAM. Participating in a lecture is associated with higher education and health literacy, which both are associated with higher interest in CAM.

Dropout rates also suggest that the questionnaire was too extensive or in other ways inconvenient for patients to complete. This may have caused another, less determinable selection bias in addition to the specific group of participants. The predetermined selection of carcinogens may have presented another problem in that it was not exhaustive and could not account for aetiological concepts of a more complex/interactive nature.

### Conclusion

Cancer patients interested in and/or using CAM cannot be assumed to have higher self-efficacy, and characteristics of these patients should be explored further in future research. If our assumption on lay-aetiological concepts being largely different from scientific ones can be confirmed even for this select group of patients, the gap might be even stronger in less educated patients. While physicians inform patients on concepts and correlations based on scientific evidence, the question arises how well this information is received and processed by the lay patient and how well-informed decisions on treatment are made. It might well be that patients facing a deadly disease like cancer give “informed consent” without being truly informed. Information material and communication must urgently be tailored to the needs of lay-people, and any communication in cases of chronic and/or serious disease should start with talking about “what is…” and “why me?”.

### Practice implications

Implications for clinical practice especially concern patient-physician-communication in cancer care. Understanding the underlying mechanisms of carcinogenesis helps patients understand modern multi-modal treatment strategies, targeted or immunological therapy. It also helps to understand concepts of neo-adjuvant or adjuvant treatment, combinations of treatments, and treatment having to continue for months and even years. Understanding is an important prerequisite for adherence and coping. In fact, the presented exploratory data suggest a tendency for patients rating carcinogenic influence in line with scientific results to have a higher self-efficacy and lead a healthier lifestyle. To improve patients’ understanding, however, it is essential that clinicians gain an idea of which aspects they need to explain in each particular case. To do that, clinicians will in turn need an understanding of the patient’s aetiological concept. Our data imply that there may be tendencies towards different “types” of concepts; however, the details of these concepts tend to be highly individualistic, emphasizing the importance of broaching the subject with a patient and encouraging them to talk about their own specific beliefs.

## Data Availability

The dataset generated and analysed during the current study are available from the corresponding author on reasonable request.
